# Risk of Low Energy Availability, Disordered Eating, Exercise Addiction, and Food Intolerances in Female Endurance Athletes

**DOI:** 10.3389/fspor.2022.869594

**Published:** 2022-05-03

**Authors:** Ida Lysdahl Fahrenholtz, Anna Katarina Melin, Paulina Wasserfurth, Andreas Stenling, Danielle Logue, Ina Garthe, Karsten Koehler, Maria Gräfnings, Mia Beck Lichtenstein, Sharon Madigan, Monica Klungland Torstveit

**Affiliations:** ^1^Department of Sport Science and Physical Education, University of Agder, Kristiansand, Norway; ^2^Department of Sport Science, Linnaeus University, Kalmar, Växjö, Sweden; ^3^Department of Sport and Health Sciences, Technical University of Munich, Munich, Germany; ^4^Department of Psychology, Umeå University, Umeå, Sweden; ^5^Sport Ireland Institute, National Sports Campus, Dublin, Ireland; ^6^The Norwegian Olympic and Paralympic Committee and Confederation of Sport, Oslo, Norway; ^7^Department of Medical Science, Dalarna University, Falun, Sweden; ^8^Centre for Telepsychiatry, Mental Health Services in the Region of Southern Denmark, Department of Clinical Research, University of Southern Denmark, Odense, Denmark

**Keywords:** Low Energy Availability in Females Questionnaire, compulsive exercise, endurance training, Relative Energy Deficiency in Sport (RED-S), eating disorder, restrictive eating behavior

## Abstract

Relative energy deficiency in sport (RED-S) is a complex syndrome describing health and performance consequences of low energy availability (LEA) and is common among female endurance athletes. Various underlying causes of LEA have been reported, including disordered eating behavior (DE), but studies investigating the association with exercise addiction and food intolerances are lacking. Therefore, the aim of this cross-sectional study was to investigate the association between DE, exercise addiction and food intolerances in athletes at risk of LEA compared to those with low risk. Female endurance athletes, 18–35 years, training ≥5 times/week were recruited in Norway, Sweden, Ireland, and Germany. Participants completed an online-survey comprising the LEA in Females Questionnaire (LEAF-Q), Exercise Addiction Inventory (EAI), Eating Disorder Examination Questionnaire (EDE-Q), and questions regarding food intolerances. Of the 202 participants who met the inclusion criteria and completed the online survey, 65% were at risk of LEA, 23% were at risk of exercise addiction, and 21% had DE. Athletes at risk of LEA had higher EDE-Q and EAI scores compared to athletes with low risk. EAI score remained higher in athletes with risk of LEA after excluding athletes with DE. Athletes at risk of LEA did not report more food intolerances (17 vs. 10%, *P* = 0.198), but were more frequently reported by athletes with DE (28 vs. 11%, *P* = 0.004). In conclusion, these athletes had a high risk of LEA, exercise addiction, and DE. Exercise addiction should be considered as an additional risk factor in the prevention, early detection, and targeted treatment of RED-S among female endurance athletes.

## Introduction

The syndrome Relative Energy Deficiency in Sport (RED-S) describes impairments of multiple physiological functions including energy metabolism, reproductive function, bone health, immune function, protein synthesis, and cardiovascular health (Mountjoy et al., 2014, 2018). The etiology behind this syndrome is low energy availability (LEA), which can occur with or without disordered eating behavior or eating disorders (Mountjoy et al., 2014, 2018). Psychological factors can therefore precede RED-S, but LEA may also result in significant psychological distress (Mountjoy et al., 2014; Ackerman et al., 2019; Langbein et al., 2021; Rogers et al., 2021). LEA seems to affect both males and females, elite athletes and recreational exercisers (Torstveit and Sundgot-Borgen, 2005; Mountjoy et al., 2014, 2018), able-bodied athletes and para-athletes (Brook et al., 2019) and across all age groups (Sharps et al., 2021).

Although any athlete can suffer from RED-S, athletes participating in weight-sensitive sports (i.e., endurance sports, combat sports, and aesthetic sports) seem to be particularly at risk. Endurance athletes also have high exercise energy expenditure (Sjödin et al., 1994) as an additional risk factor for LEA (Burke et al., 2018). Furthermore, some studies report gender differences in the sensitivity of LEA as well as the endocrine and metabolic responses, indicating a vulnerability of LEA related to females compared to males (Koehler et al., 2016). Therefore, female endurance athletes seem to be a high-risk group for RED-S.

In female endurance athletes, the reported prevalence of LEA ranges between 8 and 56% when defined as energy availability <30 kcal/kg fat-free mass/day assessed with food and training records (Monyeki et al., 2014; Melin et al., 2015; Day et al., 2016; Muia et al., 2016; Heikura et al., 2017; Mccormack et al., 2019; Beermann et al., 2020). It has been suggested that assessing self-reported physiological symptoms of LEA using questionnaires, such as the Low Energy Availability in Females Questionnaire (LEAF-Q), provides a better assessment of the overall health status of an athlete than a snapshot of current energy availability using error-prone assessments of dietary energy intake and exercise energy expenditure (Heikura et al., 2017; Burke et al., 2018; Sim and Burns, 2021). When assessed as a LEAF-Q score ≥ 8, the LEA risk rate ranges from 31 to 80% in female endurance athletes (Melin et al., 2014; Folscher et al., 2015; Heikura et al., 2017; Carr et al., 2019; Ihalainen et al., 2021; Jesus et al., 2021).

Sustained research on factors associated with LEA is important for practitioners and other health professionals for early detection and aid in the nutrition interventions to prevent potential decrements in health and performance (Melin et al., 2019; Ackerman et al., 2020). The association between disordered eating behavior and health consequences as a result of LEA in female athletes is well-researched and suggest that particular athletes from weight-sensitive sports, including endurance sports, may be at higher risk (Gibbs et al., 2013). While the prevalence of disordered eating behavior, which may lead to LEA, is reported to be higher among athletes competing in weight-sensitive sports compared with controls (Torstveit et al., 2008; Wasserfurth et al., 2020), LEA without disordered eating behavior is also common among female endurance athletes (Melin et al., 2016).

Another potential reason for LEA might be the exclusion or avoidance of different foods due to food allergies or intolerances. Although self-reported incidences of food intolerances and adherence to special diets are commonly reported in athletes (Lis et al., 2015, 2019; Logue et al., 2019), associations with LEA and RED-S among female endurance athletes have not been examined yet.

High levels of exercise energy expenditure without a corresponding increase in energy intake can result in LEA (Burke et al., 2018). It has been proposed that athletes with propensity to exercise addiction, characterized as excessive exercise behavior with potential negative consequences such as injuries and mental health problems (Griffiths et al., 2005; Lichtenstein et al., 2017), could increase the vulnerability for RED-S (Turton et al., 2017). We have previously reported an association between symptoms of exercise addiction and markers of RED-S in male endurance athletes (Torstveit et al., 2019). However, research investigating the association between exercise addiction and symptoms of RED-S among female endurance athletes is scarce with only one study investigating this association in a group representing 63% female endurance athletes (Kuikman et al., 2021). This study reported an exacerbated risk of LEA when disordered eating is accompanied with exercise addiction compared to disordered eating in isolation (Kuikman et al., 2021).

Therefore, the aim of the present cross-sectional study was to identify the risk of LEA and associated risk factors in a multi-country cohort of competitive female endurance athletes. Specifically, we aimed to compare disordered eating behavior, exercise addiction, and food intolerances in athletes at risk of LEA vs. low risk of LEA. Finally, we aimed to assess explanatory variables for the risk of LEA in this cohort. We hypothesized that athletes with risk of LEA would report more disordered eating behavior, exercise addiction, and food intolerances, have lower BMI and higher training volume compared to athletes with low risk of LEA.

## Methods

The present analysis is based on cross-sectional data collected during the screening and inclusion phase of an international multicenter intervention study aiming to induce health behavior change and improve nutritional status in female endurance athletes with symptoms of RED-S.

### Recruitment

Recruitment and data collection took place during the COVID-19 pandemic. Participants were recruited *via* Norwegian, Swedish, Irish, and German endurance competitive clubs, the Norwegian Olympic Sport Centre, Sport Ireland Institute, Swedish Olympic Committee, German Ski Federation, German Olympic Sport Confederation, and social media with a link to the project website and an online survey. Participants had to be 18–35 years of age, competitive female endurance athletes from cycling, long-distance running, orienteering, triathlon, biathlon, or cross-country skiing, training ≥5 times a week. The study was approved by the regional ethics committee in Norway (31640), Sweden (2019-04809), and Norwegian Centre for Research Data (968634). Because data collection occurred remotely and included no medical procedures, the study was considered exempt from additional approval at the other study sites. Regardless, the study was conducted in full accordance with the Declaration of Helsinki at all sites. All data were stored and analyzed in Services for Sensitive Data (University of Oslo, 2022).

### Online Survey

All participants provided written consent to their participation before they were given access to the survey. The survey questions concerned the participants' background information, including current and past sports participation, level of competition, best competition results, education attainment, occupation, training volume, age, height, body weight, menstrual dysfunction diagnosis, and food intolerances. This was followed by the validated instruments LEAF-Q (Melin et al., 2014), Exercise Addiction Inventory (EAI) (Terry et al., 2004), Eating Disorder Evaluation Questionnaire (EDE-Q) (Fairburn and Beglin, 1994), two self-constructed questions regarding history of eating disorders, and concluded with a comment section.

#### Low Energy Availability in Females Questionnaire

By assessing injury frequency, the past year, current gastrointestinal function, and current and past reproductive function the LEAF-Q was used to consider LEA related symptoms. The LEAF-Q has been validated in female endurance athletes with Cronbach's Alpha = 0.61–0.79 (Melin et al., 2014). A total score ≥8 was used to classify athletes at risk of LEA (Melin et al., 2014). Minor clarifications from the original LEAF-Q were added to question **A2:1** [“*Specify how old you were when you started taking oral contraceptives and for how long? (Months or years in total*)”], **C6** [added answer option: “*0-4 weeks*” (scoring 0 points), to the answer option “*I am pregnant…,”* “…/*I am breastfeeding*…” was added] and **D** [“…/*breastfeeding”* following *pregnancy*]. These additions do not affect the scoring key and were approved by the first author (AKM) of the development and validation of the LEAF-Q.

#### Eating Disorder Evaluation Questionnaire

The EDE-Q 6.0 was used to measure behavioral and cognitive symptoms of eating disorders the past 28 days (Fairburn and Beglin, 1994). The EDE-Q is based on the Eating Disorder Examination Interview which is considered as the gold standard in eating disorder assessment (Guest, 2000) and is one of the most used instruments to screen for eating disorder symptoms and risk of LEA/RED-S (Sim and Burns, 2021). It consists of 28 items which were divided into four subscales (restraint, eating concern, shape concern, and weight concern) and a global score averaging the subscales, used as cut-off for eating disorder pathology. A global EDE-Q score ≥ 2.5 was used to classify athletes with disordered eating behavior (Rø et al., 2015; Kuikman et al., 2021). The EDE-Q has been validated in an athletic population with Cronbach's Alpha coefficients ranging from 0.81 to 0.91 in the subscales (Lichtenstein et al., 2021a).

#### Self-Constructed Questions About Eating Disorders

Based on previous studies investigating disordered eating behavior in female athletes (Sundgot-Borgen and Torstveit, 2004), the EDE-Q was followed by two self-constructed questions regarding eating disorder history: “*Have you ever been diagnosed with an eating disorder?”* If the participants answered “*yes*,” the following question was “*What eating disorder(s) have you been diagnosed with*?” with the answer options “*Anorexia Nervosa*,” *Bulimia Nervosa*” “*Binge Eating Disorder*,” or “*Eating Disorder Not Otherwise Specified/Other Specified Feeding or Eating Disorders (e.g., atypical Anorexia or Bulimia Nervosa)*” (multiple answers allowed). If the participants' answered “*no*” to the first question, the following question was “*Do you think you have had an eating disorder even though you have not been diagnosed?”* with the following answer option: “*yes*,” “*no*,” or “*I do not know*.”

#### Exercise Addiction Inventory

The EAI was used to assess the risk of exercise addiction (Terry et al., 2004), since this tool is suggested to be more appropriate to screen the risk of exercise addiction in specific populations of exercisers compared to the other frequently used screening instrument, i.e., the Exercise Dependence Scale (Di Lodovico et al., 2019). It consists of six general components describing the degree of addiction rated on a five-point Likert scale: salience (exercise is the most important thing in life) conflicts (e.g., interpersonal conflicts due to the exercise behavior), mood modification (a coping strategy to regulate emotions), tolerance (increasing amounts of exercise is needed to achieve effect), withdrawal symptoms (e.g., irritability when a exercise session is missed), relapse (reversions to earlier patterns). Risk of addiction was defined as an EAI score ≥24 (Griffiths et al., 2005). The EAI was originally validated in recreational exercisers and has later been validated in elite athletes with a Cronbach's Alpha = 0.72 (Lichtenstein et al., 2021b). Athletes were classified with primary exercise addiction (EAI score ≥24 and EDE-Q global score <2.5) and secondary exercise addiction (EAI score ≥24 and EDE-Q global score ≥2.5).

#### Menstrual Dysfunction Diagnosis

Participants were asked: “*Do you have any diagnosis related to menstruation? [For example, polycystic ovary syndrome (PCO/PCOS)]?* With the possibility to answer “*yes*” or “*no*.”

#### Food Intolerances

To measure food intolerances or allergies, participants were asked: “*Do you have any food intolerance or allergy?*.” If the participants answered “*yes*” they were asked to answer the following question: “*Please specify your food intolerance(s)/allergy*.” To ease the readability in the paper reported food intolerances and/or allergies are here collectively termed *food intolerances*.

The online survey was pilot tested in a group of ten females who were current or former endurance athletes and subsequently adjusted where needed (minor clarifications). The survey took ~15 mins to complete. A total of 208 athletes answered the survey. After exclusion based on non-endurance sport (badminton, *n* = 1), age (*n* = 2 <18 years, *n* = 1 > 35 years), and sex (*n* = 2), a total of 202 responses were eligible for analysis.

#### Body Mass Index

Body mass index (BMI) was calculated as weight (kg) divided by height squared (m^2^). Low BMI was defined as BMI <18.5 kg/m^2^ as recommended when screening athletes for risk of LEA (Joy and Nattiv, 2017).

### Statistical Analysis

Statistical analysis was undertaken using STATA software version 16.0 (StataCorp, College Station, TX 77845, USA) with a two-tailed significance level of <0.05. Histograms were used to verify normality of distribution of continuous variables. Data are presented as mean ± standard deviation (SD) for normally distributed data and as median and interquartile range (IQ 25 and IQ 75 percentiles) for non-normally distributed values. For normally distributed data, comparisons between two independent groups were made using unpaired Student's *t*-test. To test for equality of variances, Levene's Test was applied. For non-normally distributed data, the Wilcoxon rank-sum test was used to compare two independent groups. The chi-square test for independence was used to test for differences between categorical outcomes between two independent groups. Pearson's correlation coefficient was calculated to explore associations between continuous variables for normal distributed data, while Spearman's correlation coefficient was calculated for non-normally distributed data. Finally, logistic regression models using Firth's bias reduction method (Firth, 1993; Heinze and Schemper, 2002) was used to explore possible risk factors of LEA defined as a LEAF-Q score ≥ or <8 as the dependent variable. Odds ratios and confidence intervals were used to examine associations in the logistic regression model. The Wald χ^2^ test and its accompanying *P*-value were used to examine model fit.

## Results

### Subject Characteristics and Risk of Low Energy Availability

Subject characteristics are presented in Table 1. Endurance athletes from Norway (*n* = 57), Sweden (*n* = 83), Ireland (*n* = 17), and Germany (*n* = 45) were included from the following endurance disciplines: running (*n* = 54), orienteering (*n* = 18), triathlon (*n* = 50), cycling (*n* = 45), cross country skiing (*n* = 15), and biathlon (*n* = 20). In total 65.0% of the participants were categorized as being at risk of LEA (running: 85.2%, orienteering: 77.8%, triathlon: 50.0%, cycling: 57.8%, cross country skiing: 53.8%, biathlon: 65.0%). Athletes at risk of LEA had a lower body weight and BMI compared to athletes with low risk of LEA (Table 1). There were no differences in age, height, training volume, level of competition or education comparing athletes with risk of LEA and low risk of LEA. Nor was there a difference in the risk rate of LEA between the countries (Norway: 73.7%, Sweden: 67.5%, Ireland: 64.7%, Germany: 51.1%, *P* = 0.114).

**Table 1:** Description of subjects characterized by energy availability status.

	**All**	**LEAF-Q score <8**	**LEAF-Q score ≥8**	*P*-value^*^
	***n* = 202**	***n* = 70**	***n* = 132**	
Age (y)	25 (21–30)	24 (19–30)	26 (21–30)	0.323
Height (cm)	169.4 ± 6.3	168.8 ± 5.6	169.6 ± 6.6	0.394
Body weight (kg)	61.2 ± 6.9	62.9 ± 6.9	60.3 ± 6.7	0.010
BMI (kg/m^2^)	21.3 ± 2.0	22.1 ± 2.2	20.9 ± 1.8	<0.001
BMI <18.5 kg/m^2^ (%)	7.9	4.3	9.9	0.164
Training volume (h/month)	48.2 ± 19.7	45.3 ± 19.5	49.8 ± 19.8	0.119
Full time athlete (%)	20.8	21.4	20.5	0.871
**Level of competition**				
Club (%)	66.4	65.7	66.7	0.994
National team (%)	13.4	14.3	12.9	
Professional (%)	14.4	14.3	14.4	
Other (%)	5.9	5.7	6.1	
**Level of education**				
Primary school (%)	2.0	4.3	0.8	0.248
Secondary school (%)	32.2	31.4	32.6	
University/college <4 years (%)	32.2	27.1	34.9	
University/college ≥ 4 years (%)	33.7	37.1	31.8	

*BMI, Body Mass Index; LEAF-Q, Low Energy in Females Questionnaire; LEAF-Q score ≥ 8 indicates risk of LEA.*

* *Difference between athletes at risk of LEA and low risk of LEA*.

All correlations with LEA and potential risk factors for LEA are offered in Supplementary Table S1.

Twenty-five percent of the participants reported menarche at 15 years of age or older and two had never menstruated (current age 19 and 25 years). Twenty-nine percent (*n* = 58) reported using hormonal contraceptives of which 9.1% reported using hormonal contraceptives to avoid amenorrhea. After excluding all hormonal contraceptive users, 62% were at risk of LEA. Among non-hormonal contraceptive users, 25.7% reported not having normal menstruation, while 11.8%% answered that they did not know whether their menstruation was normal or not. Of the 62.5% who reported normal menstruation among non-hormonal contraceptive users, 18.7% reported irregular periods. Thirty-three percent of the non-hormonal contraceptive users reported menstruation stoppage when exercise intensity, frequency, or duration increased. There was no difference in LEAF-Q score when comparing hormonal contraceptive users with non-users (10.2 ± 5.2 vs. 9.4 ± 4.9, *P* = 0.309), and therefore hormonal contraceptive users were included in the same manor when categorization of being at risk of LEA vs. low risk. Among all participants, two reported having a diagnosed menstrual dysfunction (polycystic ovary syndrome and unspecified secondary amenorrhea).

### Disordered Eating Behavior

As presented in Table 2, a total of 43 athletes (21.3%) had disordered eating behavior with a higher frequency among those at risk of LEA compared with those with low LEA risk (26.5 vs. 11.4%, *P* = 0.013). Athletes with disordered eating behavior had higher LEAF-Q total score compared to athletes without disordered eating behavior (12.6 ± 5.8 vs. 8.9 ± 4.4, *P* < 0.001), due to a higher gastrointestinal function score (4.0 ± 2.4 vs. 1.9 ± 1.3, *P* < 0.001). There was a positive correlation between EDE-Q score and BMI (*r* = 0.22, *P* = 0.002) and LEAF-Q score (*r* = 0.37, *P* < 0.001). All EDE-Q subscales correlated with total LEAF-Q score (restraint: *r* = 0.287, *P* < 0.001, eating concern: *r* = 0.297, *P* < 0.001, shape concern: *r* = 0.280, *P* < 0.001, weight concern: *r* = 0.284, *P* < 0.001).

**Table 2:** Symptoms of disordered eating behavior characterized by energy availability status.

	**All**	**LEAF-Q score <8**	**LEAF-Q score ≥8**	*P*-value^*^
	***n* = 202**	***n* = 70**	***n* = 132**	
**EDE-Q scales**				
Restraint	0.6 (0.2–2.2)	0.5 (0.0–1.2)	0.9 (0.2–2.6)	0.003
Eating concern	0.6 (0.2–1.4)	0.4 (0.2–0.8)	0.7 (0.2–1.8)	0.005
Shape concern	1.5 (0.8–3.0)	1.3 (0.5–2.3)	1.8 (1.0–3.3)	0.004
Weight concern	1.4 (0.6–2.6)	1.0 (0.4–2.0)	1.6 (0.6–2.9)	0.004
EDE-Q global score	1.0 (0.5–2.4)	0.7 (0.3–1.5)	1.2 (0.6–2.7)	0.001
EDE-Q ≥ 2.5 (%)	21.3	11.4	26.5	0.013

*EDE-Q, Eating Disorder Examination Questionnaire; LEAF-Q, Low Energy in Females Questionnaire*.

*LEAF-Q score ≥ 8 indicates risk of LEA. EDE-Q ≥ 2.5 indicates risk of disordered eating behavior.*

* *Difference between athletes at risk of LEA and low risk of LEA*.

Of the 43 athletes with disordered eating behavior, 41.9% of participants reported a previous eating disorder diagnosis (Anorexia Nervosa: 20.9%, Bulimia Nervosa: 16.3%, Binge Eating Disorder: 4.7%, and Other EDs: 14.0%). Among the 159 athletes with low EDE-Q score, 12.0% responded they had been diagnosed with an eating disorder in the past (Anorexia Nervosa: 5.7%, Bulimia Nervosa: 2.5%, Binge Eating Disorder: 0.6%, and Other EDs: 5.7%).

### Risk of Exercise Addiction

Athletes at risk of LEA had higher EAI score compared to athletes with low risk (Table 3). In total, 23.3% of the athletes were at risk of exercise addiction with a higher LEAF-Q total score compared to athletes with low risk of exercise addiction (12.1 ± 5.6 vs. 8.9 ± 4.5, *P* < 0.001), due to a higher injury (3.1 ± 2.2 vs. 2.2 ± 2.2, *P* = 0.013) and gastrointestinal function score (3.5 ± 2.3 vs. 2.1 ± 1.8, *P* < 0.001). Among athletes with disordered eating behavior, 60.5% (*n* = 26) were at risk of exercise addiction compared to 13.2% (*n* = 21) among athletes without disordered eating behavior (*P* < 0.001). That is, 10.4% (*n* = 21) were classified with primary exercise addiction, 12.9% (*n* = 26) with secondary exercise addiction, and 8.4% (*n* = 17) had disordered without exercise addiction. The higher EAI score in athletes with risk of LEA compared to athletes with low risk remained however after excluding athletes with disordered eating behavior (20.6 ± 3.0 vs. 19.4 ± 3.1, *P* = 0.017). After the exclusion of athletes with disordered eating behavior, 71.4% with risk of exercise addiction were also at risk of LEA compared to 59.4% without risk of exercise addiction (*P* = 0.293).

**Table 3:** Symptoms of exercise addiction characterized by energy availability status.

**Exercise addiction inventory items**	**All**	**LEAF-Q score <8**	**LEAF-Q score ≥8**	*P*-value^*^
	***n* = 202**	***n* = 70**	***n* = 132**	
Salience	3.7 ± 1.0	3.5 ± 1.0	3.9 ± 0.9	0.019
Conflicts	2.7 ± 1.3	2.3 ± 1.1	2.9 ± 1.4	0.002
Mood modification	3.6 ± 1.0	3.5 ± 0.9	3.7 ± 1.0	0.434
Tolerance	4.0 ± 0.9	4.0 ± 0.8	4.0 ± 0.9	0.733
Withdrawal symptoms	3.6 ± 1.1	3.3 ± 1.1	3.8 ± 1.0	<0.001
Relapse	3.3 ± 1.1	3.2 ± 1.1	3.3 ± 1.1	0.700
EAI total score	20.8 ± 3.5	19.8 ± 3.3	21.4 ± 3.5	0.002
EAI ≥ 24 (%)	23.3	15.7	27.3	0.064

*EAI, Exercise Addiction Inventory*.

*EAI ≥ 24 indicates risk of exercise addiction; LEAF-Q, Low Energy in Females Questionnaire*.

*LEAF-Q score ≥ 8 indicates risk of LEA.*

* *Difference between athletes at risk of LEA and low risk of LEA*.

Total EAI score was positively correlated with the EDE-Q global score (*r* = 0.47, *P* < 0.001). Further, the EAI score was positively correlated with the LEAF-Q score (*r* = 0.32, *P* < 0.001), even after excluding athletes with disordered eating behavior (*r* = 0.21, *P* = 0.007). There was no association between training volume and EAI score.

Distribution of scores on the EAI is offered in Supplementary Table S2.

### Food-Intolerances

In total, 14.4% of the athletes responded having one or more food intolerances, with gluten and lactose intolerance being the most common, and a higher total LEAF-Q score compared to athletes not reporting food intolerances (11.3 ± 4.9 vs. 9.4 ± 4.9, *P* = 0.048) due to a higher gastrointestinal function score (3.8 ± 1.9 vs. 2.1 ± 1.9, *P* < 0.001). There was no difference in the prevalence of food intolerance between athletes at risk of LEA compared to those with low risk (16.7% vs. 10.0%, *P* = 0.198). Food intolerances were more frequent among athletes with disordered eating behavior compared to athletes without disordered eating behavior (27.9% vs. 10.7%, *P* = 0.004), and among athletes at risk of exercise addiction compared to athletes with low risk of exercise addiction (29.8 vs. 9.7%, *P* = 0.001). Food intolerances were, however, not more frequent among athletes with exercise addiction compared to athletes with low risk of exercise addiction after excluding athletes with disordered eating behavior (19.0 vs. 9.4%, *P* = 0.183). Among the 29 athletes who reported food intolerances, 38.0% (*n* = 11) had simultaneously both disordered eating behavior and were at risk of exercise addiction.

### Explanatory Variables for the Risk of LEA

Results from the logistic regression analysis are presented in Table 4. The statistically significant Wald χ^2^ test indicated a better model fit when including the predictors in the model. The logistic regression analysis showed that lower BMI (*OR* = 0.69, *P* < 0.001) and higher EDE-Q score (*OR* = 1.72, *P* = 0.001) were associated with risk of LEA. None of the other predictors had a statistically significant association with LEA.

**Table 4:** Risk factors for low energy availability.

***n*** **= 202 Wald χ^2^ = *P* < 0.001**			
**Variables**	***OR***	**95% CI**	***P*** **-value**
BMI	0.69	0.57 – 0.83	<0.001
Training volume	1.01	0.99 – 1.03	0.262
EDE-Q global score	1.72	1.22 – 2.41	0.001
EAI total score	1.03	0.93 – 1.14	0.615
Food intolerances	1.31	0.49 – 3.49	0.538

*A statistically significant P value (<0.05) indicates that excluding the predictors from the model will substantially worsen model fit (i.e., the predictors add something to the model), whereas a non-significant P-value indicates that excluding the predictors will not worsen model fit (i.e., they do not add anything to the model)*.

*BMI, Body Mass Index; EAI, Exercise Addiction Inventory; EDE-Q, Eating Disorder Examination Questionnaire; LEAF-Q: Low Energy in Females Questionnaire*.

## Discussion

The main purpose of the study was to identify the risk of LEA and associated risk factors in this group of athletes, including exercise addiction and food intolerances where research has been lacking. Specifically, we aimed to compare disordered eating behavior, exercise addiction, and food intolerances in athletes at risk of LEA and low risk of LEA. A key novel finding of the study was that athletes at risk of LEA were more likely to exhibit symptoms of exercise addiction related to salience, conflicts, and withdrawal symptoms.

By using the LEAF-Q we found that 65.0% of the athletes were at risk of LEA, which is similar to the 62.2% clinically verified LEA in female endurance athletes by Melin et al. (2014). When compared to other studies which used the LEAF-Q in female endurance athletes, Carr et al. (2019), reported a lower number (31.0%) than the present study and Jesus et al. (2021) a higher number (79.5%.) In contrast to the present study, the study by Jesus et al. (2021) recruited only female athletes from cross-county running (*n* = 83) and when comparing their reported risk rate of LEA, it is similar to the risk rate among runners (85.2%) and orienteers (77.8%) in the present study. Carr et al. (2019) also had a lower number of participants (*n* = 13) only from cross-country skiing, which may partly explain the lower reported risk rate of LEA. Of note, the current cross-sectional study served as a screening phase for an intervention study aiming to improve dietary habits in athletes at risk of RED-S. Therefore, the recruitment may have attracted athletes who were interested in improving knowledge related to sports nutrition. Conversely, others may have not participated due to fears or anxiety coming from their LEA, disordered eating behavior and/or exercise addiction.

In this study we did not directly assess energy availability, which requires assessments of body composition, energy intake, and exercise energy expenditure. Although it is well-recognized that LEA is the etiological factor underpinning the syndrome of RED-S (Mountjoy et al., 2014), several barriers prohibit the direct measurement of energy availability from being a practical and reliable option (Heikura et al., 2017; Burke et al., 2018; Fahrenholtz et al., 2018; Mountjoy et al., 2018). Questionnaires can therefore be a convenient method for screening, with the LEAF-Q (Melin et al., 2014) and the EDE-Q (Fairburn and Beglin, 1994) being the most widely used and validated questionnaires in the research of RED-S (Sim and Burns, 2021).

We emphasize that although the LEAF-Q is useful for screening purposes it cannot be used as a diagnostic tool and additional individual evaluation is necessary in intervention contexts (Melin et al., 2014; Mountjoy et al., 2018). Despite acceptable sensitivity (78%) and specificity (90%) (Melin et al., 2014), there are risks of either false positive or false negative classifications. For instance, false positive cases may be due to injuries, gastrointestinal problems, and menstrual dysfunctions not related to LEA, including acute injuries, irritable bowel syndrome, or polycystic ovary syndrome, although features of polycystic ovary syndrome have been found to be prevalent in women with functional hypothalamic amenorrhea (Carmina et al., 2018). False negative cases may occur if participants use oral contraceptives, that may mask an underlying menstrual dysfunction. Other forms of hormonal contraceptives may, on the other hand, result in absence of bleeding, which accordingly can falsely be interpreted as a menstrual dysfunction.

Of note, 12.0% answered that they did not know whether they had a normal cycle and 18.7% of those who reported normal menstruation answered that they had irregular periods. This controversy indicates that a relatively high frequency of adult female endurance athletes lacks basic knowledge of female reproduction. In addition, only two athletes reported a diagnosed menstrual dysfunction, despite a high occurrence of amenorrhea and oligomenorrhea, which may relate to a perception that menstrual dysfunction often is accepted as a natural consequence of intense training programs (Beals and Meyer, 2007) or lack of awareness about the consequences of LEA among medical professionals (Curry et al., 2015). This is concerning since the menstrual cycle is an important health marker and calls for educational interventions on menstrual cycle function for female endurance athletes but also awareness among medical professionals.

In the present study, athletes at risk of LEA had lower BMI compared to athletes with a low risk of LEA and lower BMI was identified as a risk factor in the logistic regression analysis. Similar to our findings Christo et al. (2008) reported lower BMI in female endurance athletes with amenorrhea compared to their eumenorrheic counterparts. In contrast, Vanheest et al. (2014) reported higher BMI in amenorrhoeic swimmers compared to eumenorrheic swimmers, while Melin et al. (2014, 2015) found no difference in BMI comparing female endurance athletes at risk of LEA with those with a low risk of LEA. Thus, research seems conflicting when it comes to the relationship between BMI and LEA. In addition, it is important to notice, that the vast majority of athletes at risk of LEA in the current study had a BMI within the normal range, supporting that BMI alone should not be used for screening of RED-S due to potential metabolic compensatory mechanisms as a result of LEA (Mountjoy et al., 2014).

In this study, athletes at risk of LEA had higher EAI scores compared to athletes with low risk of LEA. Our results indicate that female endurance athletes with LEA are more likely to have symptoms of exercise addiction. However, in approximately half of the cases of athletes exceeding the cut-off point, exercise addiction was accompanied with disordered eating behavior. Therefore, both dietary patterns and exercise behavior should be addressed when treatment interventions for athletes with RED-S are developed. Although exercise addiction is often associated with excessive exercise (Lichtenstein et al., 2017) that may result in LEA, we found no association between training volume and EAI score, which is consistent with previous research in athletes (Lichtenstein et al., 2021b). Nor did we find a difference in the EAI item concerning increasing exercise amount when comparing athletes at high vs. low risk of LEA. One explanation for this can be that all participants in the current study had a high training volume and frequency, since this was a part of the inclusion criteria (being a competitive endurance athletes and training at least five times per week). For competitive endurance athletes it is a natural part of their training protocol to increase exercise amount to improve performance. However, we found an association between LEA and the EAI items concerning the importance exercise is attributed in life, conflicts with family and friends, and negative feelings if an exercise session is missed. The findings suggest that these exercise addiction related behaviors are more pronounced in athletes at risk of LEA, and therefore practitioners should pay attention to these personality characteristics when screening for LEA and RED-S.

Injuries are a potential consequence of both exercise addiction (Lichtenstein et al., 2017) and LEA (Mountjoy et al., 2014) and questions related to injuries are a part of the LEAF-Q due to its association to low bone mineral density (Melin et al., 2014). We found a higher injury score in athletes at risk of exercise addiction compared to athletes with low risk of exercise addiction, which may be a part of the explanation for the association between risk of LEA and exercise addiction in this group.

As reviewed by Di Lodovico et al. (2019) endurance sports are characterized with the highest risk of exercise addiction with a weighted average of 14.2% compared to 10.2% in mixed disciplines and 3.0% in the general population, although the reported risk rate spans from 0.5 to 43.0%. The risk rate of 23.3% reported in the present study is therefore considerably higher compared to previous research. The review did not distinguish between male and female responders, although female triathletes have been found to be at higher risk of exercise addiction compared to males (Griffiths et al., 2015). Hence, one potential reason for the differences in the results may be that our studied population is at high risk of disordered eating behavior. Exercise addiction is a frequent comorbid condition in female patients with eating disorders, often termed secondary exercise addiction, and therefore, eating disorder symptoms should be considered separately in the assessment of exercise addiction (Cook and Luke, 2017). Among athletes without disordered eating behavior (*n* = 159), 13.2% (*n* = 21) were at risk of exercise addiction, suggesting a risk rate of 10.4% for primary exercise addiction for the total sample. This is higher than the 4% among female athletes reported by Kuikman et al. (2021). Beyond recruiting athletes from different sports with no upper age limit, the study by Kuikman et al. (2021) differs from the present study by using the Exercise Dependence Scale, which generally identifies a lower proportion of individuals at risk of exercise addiction compared to the EAI (Di Lodovico et al., 2019). However, the occurrence of secondary exercise addiction in the present study (13%, *n* = 26) is similar to the 13% reported by Kuikman et al. (2021).

Since the EAI was originally validated in habitual exercisers, the interpretation of the questions by athletes may differ and researchers have suggested that exercise addiction is not the same phenomenon in competitive athletes as in non-competitive athletes (de la Vega et al., 2016). However, a recent study reported adequate psychometric properties of the EAI in a sample of elite athletes, suggesting that the EAI is useful for assessing symptoms of exercise addiction in competitive athletes (Lichtenstein et al., 2021b).

Disordered eating behavior and eating disorders are a well-known risk factors for LEA (Wasserfurth et al., 2020) supported by the findings in the present study. Nevertheless, the reasons for LEA are manifold (Wasserfurth et al., 2020) and in the present study, only 26.5% of the athletes at risk of LEA had disordered eating behavior, supporting the findings of Melin et al. (2014) where 28.6% of the female endurance athletes with a total LEAF-Q ≥8 had a clinically verified eating disorder or disordered eating behavior. Our results suggests that for the majority of the athletes, LEA is due to unintentional origins such as suppression of appetite post exercise (Larson-Meyer et al., 2012; Howe et al., 2016), low energy-dense diets (Melin et al., 2015), lack of knowledge regarding optimal sports nutrition (Benardot, 2013; Trakman et al., 2016; Heikkilä et al., 2018; Sim and Burns, 2021), lack of knowledge about the consequences of LEA (Folscher et al., 2015; Condo et al., 2019; Tosi et al., 2019; Logue et al., 2020) or a busy lifestyle with frequent traveling where lack of time and access to food become important barriers to adequately fueling (Benardot, 2013; Burke et al., 2018; Logue et al., 2021). Nevertheless, disordered eating behavior was common among this group of female endurance athletes with a risk rate of 21.3% using a EDE-Q global score 2.5 as cut-off compared to 24-25% earlier reported in elite female endurance athletes (Sundgot-Borgen and Torstveit, 2004; Melin et al., 2015) using the gold standard EDE Interview (Fairburn and Beglin, 1994). Collectively, these results suggest a concerning and persistently high prevalence of disordered eating among female endurance athletes.

Another potential origin to LEA could be food intolerances, since the risk of energy deficiency increases when food groups are removed from the diet, if proper replacements are not made (Lis et al., 2019). However, in contrast to our hypothesis, we did not find any differences of the frequency of reported food intolerances when comparing athletes at risk of LEA with athletes at low risk. This may indicate that athletes who report food intolerances are already familiar with finding dietary alternatives to compensate for potential deficiencies. We did, however, find a higher LEAF-Q total score among athletes reporting food intolerances, which was due to a higher gastrointestinal score. Since the study is based on self-reported data, we can only speculate whether the gastrointestinal problems are due to food intolerances or due to the gastrointestinal alterations as a result of LEA, where the symptoms may have been misinterpreted by some athletes.

Food intolerances were, however, more frequently reported by athletes with disordered eating behavior compared to athletes with low EDE-Q score, and also among athletes with risk of exercise addiction compared to athletes with low risk of exercise addiction. While there is evidence to suggest that a diagnosed food allergy increases the likelihood of developing a subsequent eating disorder (Jafri et al., 2021), it is also possible that the fear and anxiety regarding food and eating in a pre-existing eating disorder may lead to self-diagnosis to gain social acceptance for food exclusion. Likewise, the association in the present study between exercise addiction and food intolerances can potentially be explained by increased attention to the body and its reactions in athletes at risk of exercise addiction, since exercise addiction may develop as a way of dealing with difficult emotions and low self-esteem where one tries to get better mentally by finding simple physical explanations (Wågan et al., 2021). Therefore, some athletes, specifically those with secondary exercise addiction, may use food elimination as a part of their coping strategy.

### Strength and Limitations

The present study was conducted in female endurance athletes, which is the population in which the LEAF-Q was initially validated. In addition, inclusion of athletes from four different European countries increases the generalizability of the findings. To our knowledge, it is the first study to investigate the association between food intolerances and risk of LEA in female endurance athletes and one of few (Kuikman et al., 2021) to investigate the association between exercise addiction and LEA in female endurance athletes.

Studies based on self-reported data are vulnerable to response bias, denial, and inaccurate reporting, for instance when it comes to anthropometric data. However, evidence suggests that endurance athletes may more accurately self-perceive and/or report their anthropometric characteristics compared to the general population (Nikolaidis and Knechtle, 2020) and a systematic review and meta-analysis concluded that the magnitude of which self-reported data over- or underestimated the real value by women of reproductive age is negligible regarding clinical and research use (Seijo et al., 2018). Concerning the data of food intolerances, previous studies have reported a high adherence to special diets by athletes (Lis et al., 2016, 2019; Logue et al., 2019) despite lack of medical rationale (Lis et al., 2015) and it is a limitation that we did not ask whether participants who reported food intolerances had a medical verification or not. Additionally, although the EDE-Q is frequently used for screening in larger samples, it was originally developed for clinical use. The validity of the EDE-Q in athletes needs to be investigated in future studies.

We acknowledge that analysis of biomarkers of RED-S from blood samples, e.g., cortisol and triiodothyronine (Elliott-Sale et al., 2018) and measurements of bone health and resting metabolic rate (Mountjoy et al., 2018) would have strengthened the validity of the study. Unfortunately, this was not possible due to the COVID-19 pandemic. On the other hand, the self-reported data assessment enabled recruitment of athletes from all over Norway, Sweden, Ireland, and Germany, which often is problematic if participants have to meet in the laboratory.

Finally, the recruitment during the COVID-19 pandemic, may somewhat have affected the athletes' training and eating habits (Roberts et al., 2020; Shaw et al., 2021; Washif et al., 2021). The long period of isolation could potentially result in a more restricted eating behavior and/or excessive training habits for some athletes, but it is also possible that the pandemic for others have resulted in reduced training and/or increased food intake.

## Conclusion

This study confirms that female endurance athletes are at high risk of LEA (65%) and disordered eating behavior (21%), and that an association between the two exist. However, athletes with risk of LEA were also more likely to exhibit symptoms of exercise addiction and a high risk of both primary (10.4%) and secondary (13%) exercise addiction was found. In a multivariate analysis, however, symptoms of exercise addiction were not identified as predictor of LEA, nor were food intolerances, whereas lower BMI and symptoms of disordered eating behavior were. More studies are needed to investigate food intolerances and the role of exercise addiction with and without disordered eating behavior and associations with LEA among female endurance athletes.

## Data Availability Statement

The raw data supporting the conclusions of this article will be made available by the authors, without undue reservation.

## Ethics Statement

The study was approved by the Regional Ethics Committee in Norway (31640), Sweden (2019-04809), and Norwegian Centre for Research Data (968634). Because data collection occurred remotely and included no medical procedures, the study was considered exempt from additional approval at the other study sites. Regardless, the study was conducted in full accordance with the Declaration of Helsinki at all sites. All data were stored and analyzed in Services for Sensitive Data. The patients/participants provided their written informed consent to participate in this study.

## Author Contributions

ILF, AKM, IG, and MKT were responsible for study design and conceptualization. AKM and MG were responsible for the Swedish cohort. ILF, MKT, and IG the Norwegian cohort. DL and SM for the Irish cohort. PW and KK for the German cohort. ILF was responsible for coordination and overall data collection, for data analyzation under the supervision of AS, and for constructing the article where everyone reviewed and gave feedback for improvements. All authors reviewed and approved the final version of the article.

## Funding

This work was supported by Grants from the University of Agder and the Norwegian Olympic Sports Center.

## Conflict of Interest

The authors declare that the research was conducted in the absence of any commercial or financial relationships that could be construed as a potential conflict of interest.

## Publisher's Note

All claims expressed in this article are solely those of the authors and do not necessarily represent those of their affiliated organizations, or those of the publisher, the editors and the reviewers. Any product that may be evaluated in this article, or claim that may be made by its manufacturer, is not guaranteed or endorsed by the publisher.
