# Supplementary material for: Risk of Low Energy Availability, Disordered Eating, Exercise Addiction, and Food Intolerances in Female Endurance Athletes
Source: Front Sports Act Living. 2022 May 3;4:869594. doi: 10.3389/fspor.2022.869594 (PMC9110838; doi:10.3389/fspor.2022.869594)
Supplement: Supplementary file 1 [file Table_1.DOCX]

Supplementary Material

|  | LEAF-Q score | Training volume | BMI | EDE-Q global | EDE-Q restraint | EDE-Q eating concern | EDE-Q shape concern | EDE-Q weight concern | EAI total score | EAI salience | EAI conflicts | EAI mood modification | EAI tolerance | EAI withdrawal symptoms |
| --- | --- | --- | --- | --- | --- | --- | --- | --- | --- | --- | --- | --- | --- | --- |
| Training volume | *r* = 0.126  *P* = 0.073 |  |  |  |  |  |  |  |  |  |  |  |  |  |
| BMI | *r* = -0.253  *P* < 0.001 | *r* = -0.057  *P* = 0.435 |  |  |  |  |  |  |  |  |  |  |  |  |
| EDE-Q global | *r* = 0.366  *P* < 0.001 | *r* = 0.014  *P* = 0.842 | *r* = 0.265  *P* < 0.001 |  |  |  |  |  |  |  |  |  |  |  |
| EDE-Q restraint | *r* = 0.284  *P* < 0.001 | *r* = 0.057  *P* = 0.421 | *r* = 0.217  *P* = 0.002 | *r* = 0.837  *P* < 0.001 |  |  |  |  |  |  |  |  |  |  |
| EDE-Q eating concern | *r* = 0.297  *P* < 0.001 | *r* = -0.029  *P* = 0.682 | *r* = 0.207  *P* = 0.003 | *r* = 0.887  *P* < 0.001 | *r* = 0.701  *P* < 0.001 |  |  |  |  |  |  |  |  |  |
| EDE-Q shape concern | *r* = 0.280  *P* <0.001 | *r* = -0.006  *P* = 0.934 | *r* = 0.263  *P* < 0.001 | *r* = 0.960  *P* <0.001 | *r* = 0.728  *P* < 0.001 | *r* = 0.841  *P* < 0.001 |  |  |  |  |  |  |  |  |
| EDE-Q weight concern | *r* = 0.284  *P* < 0.001 | *r* = 0.0369  *P* = 0.602 | *r* = 0.262  *P* < 0.001 | *r* = 0.937  *P* < 0.001 | *r* = 0.679  *P* < 0.001 | *r* = 0.800  *P* < 0.001 | *r* = 0.904  *P* < 0.001 |  |  |  |  |  |  |  |
| EAI total score | *r* = 0.318  *P* < 0.001 | *r* = 0.026  *P* = 0.709 | *r* = -0.143  *P* = 0.042 | *r* = 0.439  *P* < 0.001 | *r* = 0.396  *P* < 0.001 | *r* = 0.396  *P* < 0.001 | *r* = 0.412  *P* < 0.001 | *r* = 0.365  *P* < 0.001 |  |  |  |  |  |  |
| EAI salience | *r* = 0.167  *P* = 0.018 | *r* = 0.131  *P* = 0.064 | *r* = -0.065  *P* = 0.361 | *r* = 0.179  *P* = 0.011 | *r* = 0.134  *P* = 0.058 | *r* = 0.157  *P* = 0.026 | *r* = 0.159  *P* = 0.024 | *r* = 0.201  *P* = 0.004 | *r* = 0.506  *P* < 0.001 |  |  |  |  |  |
| EAI conflicts | *r* = 0.298  *P* < 0.001 | *r* = 0.020  *P* = 0.778 | *r* = -0.126  *P* = 0.076 | *r* = 0.307  *P* < 0.001 | *r* = 0.263  *P* < 0.001 | *r* = 0.263  *P* < 0.001 | *r* = 0.289  *P* < 0.001 | *r* = 0.253  *P* < 0.001 | *r* = 0.570  *P* < 0.001 | *r* = 0.040  *P* = 0.572 |  |  |  |  |
| EAI mood modification | *r* = 0.116  *P* = 0.100 | *r* = -0.241  *P* < 0.001 | *r* = 0.001  *P* = 0.988 | *r* = 0.316  *P* < 0.001 | *r* = 0.306  *P* < 0.001 | *r* = 0.287  *P* < 0.001 | *r* = 0.306  *P* < 0.001 | *r* = 0.226  *P* = 0.001 | *r* = 0.578  *P* < 0.001 | *r* = 0.105  *P* = 0.137 | *r* = 0.188  *P* = 0.008 |  |  |  |
| EAI tolerance | *r* = 0.031  *P* = 0.659 | *r* = 0.143  *P* = 0.043 | *r* = -0.001  *P* = 0.989 | *r* = 0.008  *P* = 0.907 | *r* = 0.050  *P* = 0.482 | *r* = -0.043  *P* = 0.548 | *r* = -0.015  *P* = 0.833 | *r* = -0.010  *P* = 0.988 | *r* = 0.367  *P* < 0.001 | *r* = 0.256  *P* < 0.001 | *r* = 0.012  *P* = 0.869 | *r* = 0.023  *P* = 0.746 |  |  |
| EAI withdrawal symptoms | *r* = 0.305  *P* < 0.001 | *r* = 0.084  *P* =0.233 | *r* = -0.193  *P* = 0.006 | *r* = 0.397  *P* < 0.001 | *r* = 0.365  *P* < 0.001 | *r* = 0.362  *P* < 0.001 | *r* = 0.394  *P* < 0.001 | *r* = 0.330  *P* < 0.001 | *r* = 0.700  *P* < 0.001 | *r* = 0.291  *P* < 0.001 | *r* = 0.286  *P* < 0.001 | *r* = 0.324  *P* < 0.001 | *r* = 0.137  *P* = 0.053 |  |
| EAI relapse | *r* = 0.103  *P* = 0.142 | *r* = -0.018  *P* =0.795 | *r* = .0.046  *P* = 0.518 | *r* = 0.262  *P* < 0.001 | *r* = 0.237  *P* < 0.001 | *r* = 0.271  *P* < 0.001 | *r* = 0.260  *P* < 0.001 | *r* = 0.223  *P* = 0.002 | *r* = 0.599  *P* < 0.001 | *r* = 0.151  *P* = 0.033 | *r* = 0.191  *P* < 0.001 | *r* = 0.264  *P* < 0.001 | *r* = 0.044  *P* = 0.538 | *r* = 0.307  *P* < 0.001 |

**Table S1. Correlation table with potential risk factors for LEA**

Abbreviations: BMI: body mass index, EAI: Exercise Addiction Inventory, EDE-Q: Eating Disorder Examination Questionnaire, LEAF-Q: Low Energy in Females Questionnaire

**Table S2. Distribution of scores on the Exercise Addiction Inventory**

|  | **Strongly disagree** | | **Disagree** | | **Neither agree/disagree** | | **Agree** | | **Strongly agree** | |
| --- | --- | --- | --- | --- | --- | --- | --- | --- | --- | --- |
| **EAI items** | LEAF-Q score < 8 | LEAF-Q score ≥ 8 | LEAF-Q score < 8 | LEAF-Q score ≥ 8 | LEAF-Q score < 8 | LEAF-Q score ≥ 8 | LEAF-Q score < 8 | LEAF-Q score ≥ 8 | LEAF-Q score < 8 | LEAF-Q score ≥ 8 |
| Salience (%) | 1.4 | 0.8 | 17.1 | 7.6 | 25.7 | 20.6 | 38.6 | 47.3 | 17.1 | 23.7 |
| Conflicts (%) | 28.6 | 24.4 | 35.7 | 16.0 | 15.7 | 19.1 | 17.1 | 29.8 | 2.9 | 10.7 |
| Mood modification (%) | 1.43 | 3.0 | 17.1 | 10.6 | 17.1 | 25.8 | 54.3 | 38.4 | 10.0 | 22.0 |
| Tolerance (%) | 1.4 | 0.0 | 5.7 | 6.9 | 10.0 | 17.6 | 60.0 | 42.8 | 22.9 | 32.8 |
| Withdrawal symptoms (%) | 2.9 | 2.3 | 28.6 | 7.6 | 25.7 | 22.0 | 25.7 | 45.5 | 17.1 | 22.7 |
| Relapse (%) | 4.4 | 3.8 | 24.6 | 24.2 | 24.6 | 28.0 | 37.7 | 28.0 | 8.7 | 15.9 |

Abbreviations: EAI: Exercise Addiction Inventory, LEAF-Q: Low Energy in Females Questionnaire
